# Molecular Dynamics Insights into Mechanical Stability, Elastic Properties, and Fracture Behavior of PHOTH-Graphene

**DOI:** 10.3390/ma17194740

**Published:** 2024-09-27

**Authors:** Qing Peng, Gen Chen, Zeyu Huang, Xue Chen, Ao Li, Xintian Cai, Yuqiang Zhang, Xiao-Jia Chen, Zhongwei Hu

**Affiliations:** 1 School of Science, Harbin Institute of Technology, Shenzhen 518055, China; 2 State Key Laboratory of Nonlinear Mechanics, Institute of Mechanics, Chinese Academy of Sciences, Beijing 100190, China; 22013080001@stu.hqu.edu.cn (G.C.); 22014080032@stu.hqu.edu.cn (Z.H.); chenxue@imech.ac.cn (X.C.); ao@youzi.com (A.L.); caixintian@whu.edu.cn (X.C.); 3 Guangdong Aerospace Research Academy, Guangzhou 511458, China; 4 Institute of Manufacturing Engineering, Huaqiao University, Xiamen 361021, China; 19013080047@stu.hqu.edu.cn; 5 Institute of Mechanical Engineering and Automation, Huaqiao University, Xiamen 361021, China; 6 Hubei Key Laboratory of Electronic Manufacturing and Packaging Integration, Wuhan University, Wuhan 430072, China

**Keywords:** PHOTH-graphene, mechanical property, MD, defects, hydrogen functionalization

## Abstract

PHOTH-graphene is a newly predicted 2D carbon material with a low-energy structure. However, its mechanical stability and fracture properties are still elusive. The mechanical stability, elastic, and fracture properties of PHOTH-graphene were investigated using classical molecular dynamics (MD) simulations equipped with REBO potential in this study. The influence of orientation and temperature on mechanical properties was evaluated. Specifically, the Young’s modulus, toughness, and ultimate stress and strain varied by −26.14%, 36.46%, 29.04%, and 25.12%, respectively, when comparing the armchair direction to the zigzag direction. The percentage reduction in ultimate stress, ultimate strain, and toughness of the material in both directions after a temperature increase of 1000 K (from 200 K to 1200 K) ranged from 56.69% to 91.80%, and the Young’s modulus was reduced by 13.63% and 7.25% in both directions, respectively, with Young’s modulus showing lower sensitivity. Defects usually weaken the material’s strength, but adding random point defects in the range of 3% to 5% significantly increases the ultimate strain of the material. Furthermore, hydrogen atom adsorption induces crack expansion to occur earlier, and the crack tip without hydrogen atom adsorption just began to expand when the strain was 0.135, while the crack tip with hydrogen atom adsorption had already undergone significant expansion. This study provides a reference for the possible future practical application of PHOTH-graphene in terms of mechanical properties and fracture failure.

## 1. Introduction

Two-dimensional graphene exhibits excellent properties and is employed in many applications, including electronic components, hydrogen storage, batteries, and desalination membranes [1,2,3,4,5,6,7]. The Young’s modulus of monolayer graphene is up to 1 TPa, the ultimate stress can reach 130 GPa, and the ultimate strain can reach more than 0.20 [8,9,10,11,12]. The extraordinary mechanical properties of graphene have attracted researchers to conduct a series of studies on its mechanical properties and fracture failure mechanisms. These studies have included an investigation of the material’s anisotropy and an examination of the influences of temperature, defects, and functional group adsorption on both mechanical properties and fracture behavior. The strength of graphene is compromised at elevated temperatures [13,14], with the material exhibiting brittle fracture behavior [15]. Even at ultrahigh temperatures of 4000 K~5000 K, the fracture mode of graphene remains brittle [16]. Defects are inevitable in the preparation and engineering applications of graphene, and common forms of defects include Stone–Thrower–Wales (STW) defects, vacancy defects, crack and line defects (dislocations and grain boundaries) [17,18,19], etc. The introduction of defects usually weakens the strength of the material [20,21,22], but the mechanical properties of graphene can also be improved through reasonable design of the defects to control anisotropy and enhance toughness [17,23,24,25,26]. Carbon materials, represented by graphene, are considered to be excellent solid-state hydrogen storage materials due to their high porosity [27,28]. Hydrogen atoms in the external environment change the electronic properties and magnetic properties of the carbon atoms by altering the structure of the C-C bonds in the localized regions of the material [29,30,31,32,33,34], which ultimately causes the strength of the material to weaken [35,36,37].

The prediction of new structures for graphene derivatives using first-principles approaches has attracted scientists’ attention in recent years due to graphene’s impressive and excellent mechanical properties. The most predicted new two-dimensional carbon materials exhibit specialized structures and excellent properties, rendering them highly promising for specific engineering applications such as lithium batteries, hydrogen storage, and more [38,39,40,41,42,43]. However, there are still difficulties and challenges in preparing these new materials. To accelerate the process of designing, developing, manufacturing, and ultimately engineering these new materials, MD methods were used to test the physical characteristics of the new two-dimensional carbon materials [44,45,46,47,48]. The results of MD testing can be utilized to inform the development of novel materials and practical engineering applications, thereby accelerating the process of integrating new materials into industrial applications.

PHTOH-graphene is a recently predicted two-dimensional carbon material with an inherent low-energy structure that provides the material with good mechanical properties and thermal stability, and researchers have used the AIMD method to study its basic physical properties [49]. When studying the mechanical properties of materials on a larger scale, AIMD is no longer applicable due to its much higher computational cost compared to classical MD. Our study used the classical MD approach to investigate the mechanical stability, elastic, and fracture properties in different orientations (armchair and zigzag). It also investigated the effects of temperature, defects (random vacancy defects and type I cracks), and hydrogen atom adsorption at the crack tip on the material’s mechanical properties. This research will provide a reference for the possible subsequent practical applications of PHOTH-graphene.

## 2. Materials and Methods

**Figure 1:**
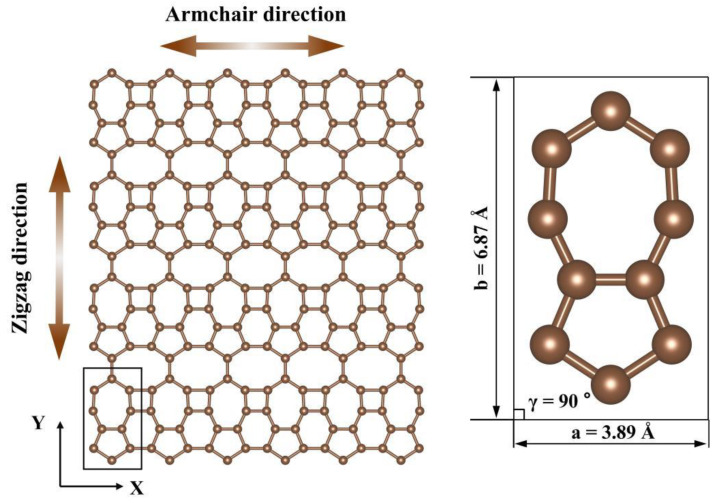
Schematic structure of PHOTH-graphene (**left**), and a primitive unit cell (**right**) with lattice constants values. All atoms are coplanar.

As illustrated in Figure 1, PHOTH-graphene comprises four-, five-, six-, seven-, and eight-membered carbon rings with ten carbon atoms in its protocells. The lattice constants a and b are labeled in Figure 1, and the C-C bonds exhibit bond lengths between 0.1410 nm and 0.1435 nm. The lattice is orthogonally aligned in the P1 space group. In the model, the X and Y directions are defined in terms of the orientation of the six-membered ring as the armchair and zigzag directions. The model utilized in this study comprises 24,800 carbon atoms, and the model’s size in the plane is 27.62×27.48 nm. Concerning the thickness of single-layer graphene, the thickness of single-layer PHOTH-graphene is set to 0.334 nm. The simulated box has a thickness of 13.74 nm in the z-direction, with single-layer PHOTH-graphene situated in the middle of the box in the z-direction. The impact of the model’s size on the simulation will be discussed in the subsequent section.

This study conducted simulations using the classical molecular dynamics method, and the simulation software selected was LAMMPS (lammps-23Jun2022) [50]. The visualization software utilizes OVITO (Version 3.7.10) [51]. The basic parameters of the simulation included a time step of 0.0001 ps and a strain rate of 0.001 ps^−1^; the temperature was maintained at 300 K throughout the simulation, except during the investigation of temperature effects.

The stress–strain relationship is considered the most significant indicator during in-plane mechanical property testing of two-dimensional carbon materials and is commonly used to evaluate the mechanical properties of two-dimensional materials [22,45,52,53]. The stress–strain curve can be used to extract some crucial pieces of information. Young’s modulus is calculated by fitting a linear function to the initial segment of the stress–strain curve. Ultimate stress and strain correspond to the points of maximum stress and strain, respectively, where failure typically occurs. Toughness is determined by integrating the area under the stress–strain curve.

The influence of defects on the fracture failure of a material is critical. It is commonly observed that failure of a material occurs preferentially at locations in the material where defects are present due to the stress concentrations that occur at these locations under load [22,45,54]. Two forms of defects are considered in this paper: random vacancy defects and type I cracks. The former is achieved by random deletion of carbon atoms, while the latter is achieved by deletion of carbon atoms in a designated region to create a certain crack shape. Type I cracks, also known as tensile cracks, are characterized by the dominance of normal tensile stress on the crack surface. The crack extension is oriented perpendicular to the applied stress. Griffith’s theory is frequently employed to describe the fracture behavior of two-dimensional carbon materials in the presence of pre-cracks. Its mathematical form is as follows [55]:

(1) $$\sigma_{f}=\sqrt{\frac{2E\gamma_{s}}{\pi a}}$$

where $\gamma_{s}$—surface free energy; E—Young’s modulus; a—crack length; and $\sigma_{f}$—fracture stress.

The fracture stress of a material is closely associated with the size of the crack. Materials with cracks typically exhibit expansion from the crack’s tip, which ultimately leads to the material’s fracture failure.

The 2nd generation REBO potential function is used to define interactions between carbon atoms [56] and is widely employed to study the physical behavior of two-dimensional carbon materials [16,57,58]. We used an optimized REBO potential function suitable for PHOTH-graphene. The details of the optimization will be discussed in the next section. The mathematical form of the REBO potential function, which describes the interaction between the C-C bond, is presented in Equation (2):

(2) $$E_{ij}^{Rebo}=f(r_{ij})[V_{ij}^{R}(r_{ij})+b_{ij}V_{ij}^{A}(r_{ij})]$$

where i and j indicate the serial numbers of two different atoms, respectively, $r_{ij}$—the distance between two atoms, $b_{ij}$—the bond order term that regulates the bond strength based on the bonding environment, $V_{ij}^{R}$—mutual repulsive interactions between atoms, $V_{ij}^{A}$—mutual attraction between atoms, and $f(r_{ij})$—cut-off function.

The cut-off function is shown in Equation (3):

(3) $$f(r_{ij})=\left\{\begin{array}{ll} \;\;\;\;1, & r_{ij}<\mathrm{rcmin}\_\mathrm{CC}\\ \frac{1+cos[\pi (r_{ij}-rcmin\_CC)/(rcmax\_CC-rcmin\_CC)]}{2}, & \mathrm{rcmin}\_\mathrm{CC}<r_{ij}<\mathrm{rcmax}\_\mathrm{CC}\\ \;\;\;\;\;\;0, & r_{ij}>\mathrm{rcmax}\_\mathrm{CC} \end{array}\right.$$

Among them, rcmin_CC and rcmax_CC are the cut-off radius of the soft cut-off, and they define the change in the cut-off function from 0 to 1. Obviously, as the interatomic distance $r_{ij}$ increases, the value of the cut-off function gradually increases from 0 to 1, which avoids an abrupt change in the cut-off function in the numerical value, and this method of taking the value of the cut-off radius is known as the soft cut-off. Material fracture is an abrupt process, and the cut-off function is set to avoid the computational problems that abrupt changes may cause. Such a practice exists in the Tersoff, REBO, and AIREBO potential functions. rcmin_CC = 1.70 and rcmax_CC = 2.0 are the defaults in the REBO potential function, and the introduction of a soft truncation makes the fracture process of the C-C bond smoother, which is helpful in capturing the physical response of the material during the fracture process. However, a soft cut-off that interferes excessively with the fracture behavior of the material results in a material that exhibits non-physical strain hardening.

To overcome the non-physical strain hardening observed in the stress–strain curve due to the cut-off radius, a hard cut-off (r = rcmin_CC = rcmax_CC = 2.0) with a cut-off radius of 2.0 has been proposed for use in the simulation of the fracture failure of 2D graphene. This approach allows for simplified calculations while avoiding the effect of an excessive soft cut-off on the fracture behavior of the material, as the C-C bond will rapidly undergo brittle fracture after reaching the ultimate strain. This methodology avoids the excessive influence of soft cut-off on the fracture behavior of the material, thereby resulting in a calculation that is more in line with the experimental results [59,60,61,62,63,64]. The cut-off function for a hard cut-off is shown in Equation (4):

(4) $$f(r_{ij})=\left\{\begin{array}{ll} 1, & r_{ij}<r\\ 0, & r_{ij}>r \end{array}\right.$$

where $r_{ij}$—distance between atoms and r—cut-off radius.

Furthermore, while maintaining the benefit of the soft cut-off of the REBO potential function, the potential function is optimized by adjusting the value of rcmin_CC (the typical values are 1.92 and 1.95). Additionally, the optimized potential function also demonstrates satisfactory outcomes [17,65]. In summary, the current solution to strain hardening of materials exhibiting non-physical behavior mainly consists of setting a hard cut-off and adjusting the cut-off radius of the soft cut-off.

Functional adsorption on two-dimensional carbon materials has a critical effect on fracture failure. The adsorption of hydrogen atoms on graphene, in particular, has been shown to result in a reduction in the mechanical properties of these materials [27,66,67]. Two-dimensional carbon materials have good prospects for applications in the direction of hydrogen storage, so a hydrogen-filled atmosphere would be a potential working environment for PHOTH-graphene. Building on the analysis of microcracks’ impact on the material’s fracture failure, we further investigated the influence of hydrogen atom adsorption at the crack tip on the material’s fracture failure.

## 3. Results and Discussion

### 3.1. Optimization of the Potential Function

The uniaxial stretching of PHOTH-graphene was carried out using the original REBO potential function in order to test its mechanical properties, and the stress–strain curves exhibited a non-physical behavior of material hardening. Based on the analysis above, we believe that this is due to excessive interference by the soft cut-off on the fracture behavior of the material. In previous studies, the convergence test on the value of the cut-off radius has been demonstrated to be an essential method for optimizing the potential function, which allows for an assessment of the value of the cut-off radius, which in turn enables the selection of a more appropriate cut-off radius. Furthermore, the optimized potential function can be employed in uniaxial tensile testing to enhance the ability to capture the mechanical response of the material behavior [68,69]. Therefore, it is necessary to modify the cut-off radius of the soft cut-off in order to use it to test the mechanical properties of PHOTH-graphene.

**Figure 2:**
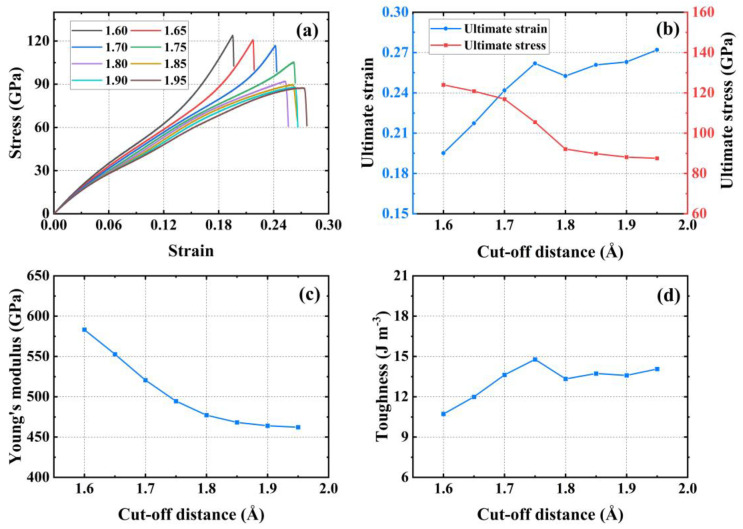
Convergence test for cut-off radius. (**a**) Stress–strain curves; (**b**) ultimate stress and strain; (**c**) Young’s modulus; (**d**) toughness.

As shown in Figure 2, we tested the convergence of the cut-off radius of the soft cut-off by keeping the value of rcmax_CC unchanged at 2.0 Å and taking rcmin_CC at 1.60 Å, 1.65 Å, 1.70 Å, 1.75 Å, 1.80 Å, 1.85 Å, 1.90 Å, and 1.95 Åy. Figure 2a shows that the stress–strain curves of uniaxial stretching exhibits the non-physical behavior of strain-hardening during the stretching process for all values of rcmin_CC that were less than 1.80 Å. As shown in Figure 2b, the ultimate stress and strain converge with the gradual increase in rcmin_CC to 1.80 Å. The ultimate stresses at rcmin_CC values of 1.60 Å, 1.65 Å, 1.70 Å, and 1.75 Å increase by 34.58%, 31.18%, 26.89%, and 14.46%, respectively, compared to the ultimate stresses at rcmin_CC of 1.80 Å. The ultimate strains at 1.85 Å, 1.90 Å, and 1.95 Å decreased by 2.48%, 4.43%, and 5.21%, respectively, compared to the ultimate stresses for rcmin_CC at 1.80 Å. Figure 2c demonstrates the variation in Young’s modulus with the cut-off radius. The Young’s modulus varies more for rcmin_CC values less than 1.80 Å. For the rcmin_CC values of 1.85 Å, 1.90 Å, and 1.95 Å, the Young’s modulus decreases by 1.89%, 2.78%, and 3.13% compared to the Young’s modulus when rcmin_CC is 1.80 Å. The Young’s modulus tends to converge for values of rcmin_CC greater than 1.80 Å (values of rcmin_CC equal 1.85 Å, 1.90 Å, and 1.95 Å) and decreases by 1.89%, 2.78%, and 3.13%, respectively. Figure 2d demonstrates the variation in toughness with the change in cut-off radius; the toughness varies more for rcmin_CC values less than 1.80 Å, and for rcmin_CC values of 1.85 Å, 1.90 Å, and 1.95 Å, the toughness increases by 2.97%, 2.01%, and 5.51% compared to the toughness when rcmin_CC is 1.80 Å. The toughness of the material tends to converge after the value of rcmin_CC is greater than 1.80 Å. The toughness of the material increases by 2.97%, 2.01%, and 5.51% when the value of rcmin_CC is greater than 1.80 Å. In summary, the mechanical properties of the material begin to converge after rcmin_CC is taken to be 1.80 Å. Therefore, rcmin_CC = 1.80 Å is taken as the soft cut-off.

**Figure 3:**
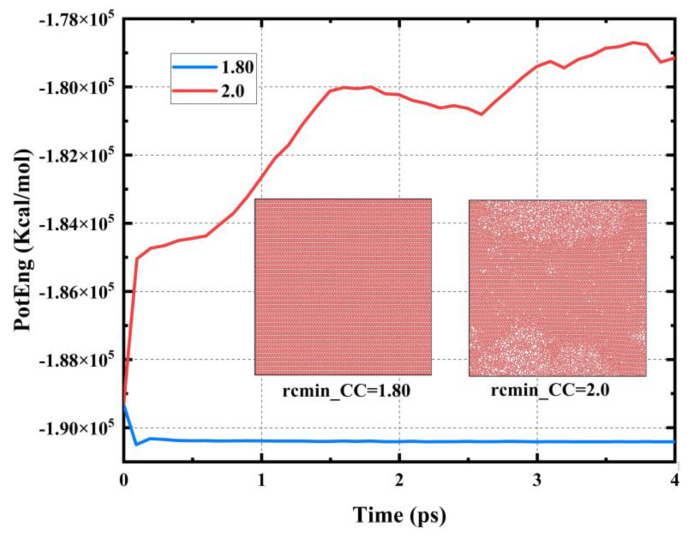
Potential energy changes in the simulated system during the relaxation process using both the hard cut-off and soft cut-off methods.

Furthermore, we also tested the applicability of the hard cut-off (rcmin_CC = rcmax_CC = 2.0 Å) for the mechanical property testing of PHOTH-graphene. Figure 3 shows the system’s potential energy change during the relaxation process. The potential energy of the system grows significantly. The structure changes significantly under the hard cut-off, whereas the soft cut-off with rcmin_CC = 1.80 Å is used for the system’s structural integrity and the potential energy of the system remains well stabilized during the relaxation process. Therefore, the hard cut-off is not suitable for testing the physical behavior of the new PHOTH-graphene material. Combining our tests on the convergence of the cut-off radius in the soft cut-off approach and the feasibility of the hard cut-off approach, we finally conclude that the soft cut-off approach is more suitable for testing the mechanical properties of PHOTH-graphene. The optimized REBO potential function with rcmin_CC = 1.80 Å and rcmax_CC = 2.0 Å was used in the subsequent studies.

### 3.2. Size Effects

**Figure 4:**
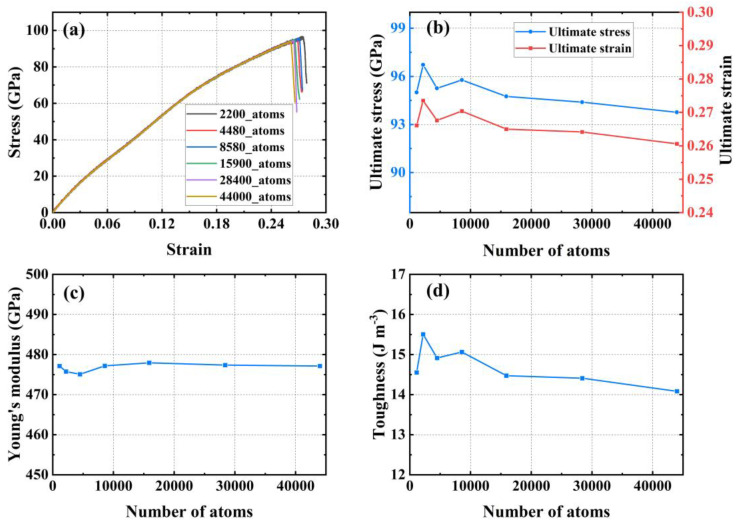
System size convergence tests. (**a**) Stress–strain curve; (**b**) ultimate stress and strain; (**c**) Young’s modulus; (**d**) toughness.

Selecting an appropriate system size can help to obtain more accurate results more economically. In general, it is difficult to obtain accurate results with too small a system of the model, and a larger system helps to obtain more accurate results. However, simply increasing the system size of the simulation shows limited improvements in the accuracy of the simulation results, so it is necessary to perform a convergence test on the system size of the simulation. As shown in Figure 4, the system size was tested for convergence using systems containing 2200, 4480, 8580, 15,900, 28,400, and 44,000 carbon atoms, respectively, along the armchair direction.

Figure 4a illustrates the stress–strain relationships from the uniaxial tensile tests for six different sized systems. Figure 4b shows the ultimate stress and strain obtained from testing systems of different sizes. As the system size increases, the values of the ultimate stress and strain tend to stabilize. The system containing 28,400 carbon atoms shows a change of −0.49% in ultimate stress and −1.99% in ultimate strain compared to the system with 15,900 carbon atoms. Figure 4c shows the Young’s modulus from uniaxial tensile testing of systems with different sizes. The Young’s modulus for the four systems, each with a number of carbon atoms greater than or equal to 8580, remains essentially the same, with a maximum difference of only 2.01 GPa. Figure 4d demonstrates the toughness of the material used in the uniaxial tensile tests conducted for systems of different sizes, and the toughness measured for the three systems with a number of carbon atoms greater than or equal to 15,900 tends to converge, with a difference of only 0.39 J m^−3^ between the maximum and minimum values. To guarantee the precision and efficacy of the calculations, we decided that subsequent studies in this paper would contain 28,400 carbon atoms in the system used for analysis.

### 3.3. Mechanical Properties

#### 3.3.1. Mechanical Properties in Different Orientations

The mechanical properties of PHOTH-graphene are listed in Table 1. The Young’s modulus is 26.14% higher in the zigzag direction compared to the armchair direction. The ultimate stress and strain in the armchair direction are also higher than in the zigzag direction by 29.04% and 25.12%, respectively. The material’s toughness is 36.46% greater in the armchair direction than in the zigzag direction. In conclusion, the material shows excellent mechanical properties in both directions.

**Figure 5:**
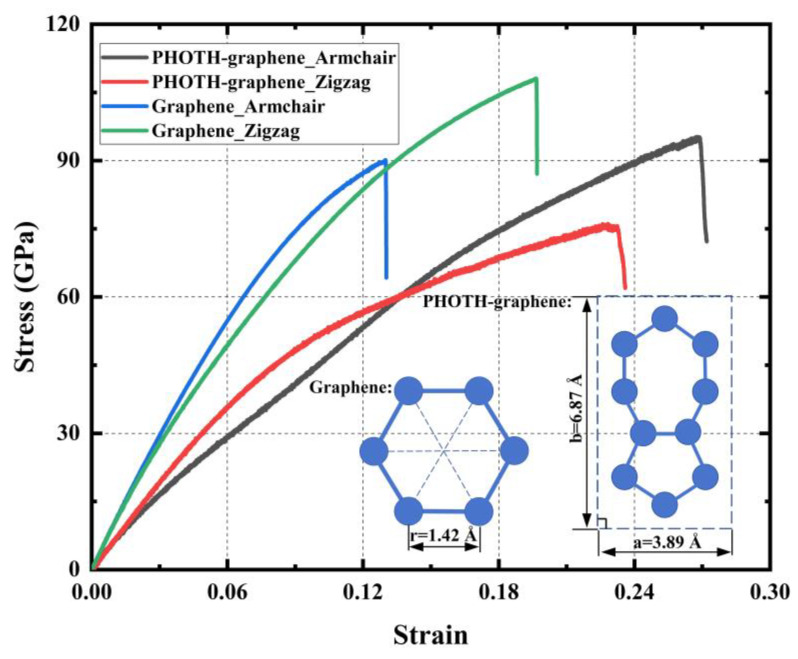
Stress–strain curves of PHOTH-graphene and graphene in different tensile directions.

Furthermore, we conducted a comparative analysis of PHOTH-graphene and graphene. The simulation of graphene was performed using the AIREBO potential function with a hard truncation, with a cut-off radius of 2.0 Å. The remaining simulation conditions remained consistent with those of PHOTH-graphene. Figure 5 shows that Young’s modulus of graphene is 964.07 GPa and 1007.99 GPa in the zigzag and armchair directions, which is consistent with earlier studies [9,69] and is considerably higher than that of PHOTH-graphene in both directions. The exceptional mechanical properties of graphene are attributed to its distinctive six-membered ring structure. To elucidate the rationale behind the disparity in the mechanical properties of graphene and PHOTH-graphene, we introduce the concept of mass surface density, which reflects the structural characteristics of two-dimensional carbon materials in the plane to a certain extent. It is commonly observed that the differing structural characteristics of two-dimensional carbon materials will result in disparate mass surface density.

The mass surface density of the two-dimensional carbon material can be calculated using Equation (5).

(5) $$\rho_{ij}=\frac{m}{S}$$

where m—the total mass of carbon atoms and S—the area of the region containing a certain number of carbon atoms.

The mass face density of graphene is calculated using Equation (5) as $7.66\times 10^{-7}\;kg/m^{2}$, while that of PHOTH-graphene is calculated as $7.46\times 10^{-7}\;kg/m^{2}$. The face density of graphene is found to be 2.7% greater than that of PHOTH-graphene, resulting in graphene exhibiting superior mechanical properties and a larger Young’s modulus. Mass face density clearly explains the difference in the mechanical properties between graphene and PHOTH-graphene.

The projected line density of a two-dimensional carbon material can be calculated from the surface density.

(6) $$\left\{\begin{array}{c} \rho_{i}=\rho_{ij}\times l_{j}\\ \rho_{j}=\rho_{ij}\times l_{i} \end{array}\right.$$

where $\rho_{ij}$—mass surface density, $\rho_{i}$—projected line density of the material along the i-direction, $\rho_{j}$—projected line density of the material along the j-direction, $l_{i}$—the length of the projection of the region along the i-direction, and $l_{j}$—the length of the projection of the region along the j-direction.

The projected linear densities of PHOTH-graphene along the armchair and zigzag directions can be calculated from Equation (6) as $5.125\times 10^{-16}\;kg/m$ and $2.902\times 10^{-16}\;kg/m$. The ultimate stress and strain along the armchair direction in the stress–strain curves are larger, which show stronger tensile toughness. However, the Young’s modulus along the armchair direction is smaller than that along the zigzag direction. This difference arises because the projected line density, unlike the mass surface density, is one-dimensional and overlooks the material’s structural characteristics along the projected direction. Since Young’s modulus is closely related to the material’s structure, this oversight leads to a lower modulus in the armchair direction.

#### 3.3.2. Forms of Failure and Fracture

**Figure 6:**
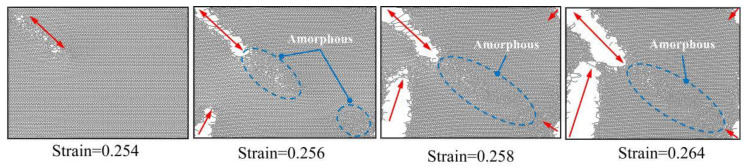
Failure fracture behavior of PHOTH-graphene under uniaxial tension along the armchair direction.

To gain further insight into the failure and fracture behavior of PHOTH-graphene, we took snapshots of the material during uniaxial stretching in both directions. As shown in Figure 6, the material shows microcracks at a strain of 0.254 in the armchair direction. By the time the strain stage reaches 0.256, the crack size has expanded rapidly, and a region containing an amorphous structure has appeared in the direction of the crack tip. As the strain increases to 0.258, the crack size expands further, and the area containing the amorphous structure increases. Eventually, the material fails and fractures when the strain reaches the ultimate strain of 0.264.

**Figure 7:**
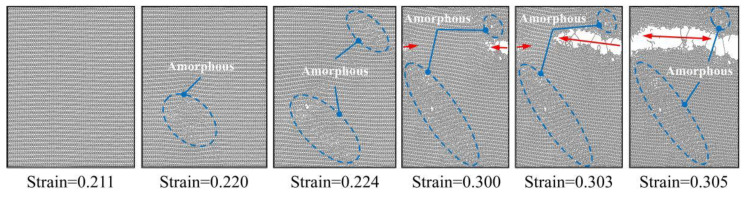
Failure fracture behavior of PHOTH-graphene under uniaxial tension along the zigzag direction.

As shown in Figure 7, the material did not show significant microcracking and phase transformation when the material reached the ultimate strain of 0.211 in the zigzag direction. Further increasing the strain to 0.220 on the basis of the ultimate strain, the material shows obvious regions containing amorphous structures. At a strain of 0.224, the area of the material containing amorphous structure increases. At a strain of 0.300, the area of the material containing the amorphous structure increases further and microcracks appear in the amorphous structure. At a strain of 0.303, the size of the cracks is rapidly expanded. At a strain of 0.305, further expansion of the crack size occurs.

The form of failure fracture differs between the armchair and zigzag directions. Microcracks are first observed during uniaxial stretching in the armchair direction, but the material does not fail entirely at this stage. As the crack expands towards the crack tip, it encounters a region with an amorphous structure. The material then reaches ultimate strain with rapid crack expansion, eventually leading to brittle fracture failure. A region containing an amorphous structure is first observed during uniaxial stretching in the zigzag direction. The appearance of the amorphous structure significantly weakens the material’s overall bearing capacity, indicating initial failure. Microcracks then appear near the amorphous region, and the cracks expand rapidly with further strain increase.

#### 3.3.3. Mechanical Properties at Different Temperatures

To investigate the influence of temperature on the mechanical properties of PHOTH-graphene, we tested the material’s properties along the armchair and zigzag directions at temperatures of 200 K, 300 K, 400 K, 500 K, 600 K, 700 K, 900 K, and 1200 K.

**Figure 8:**
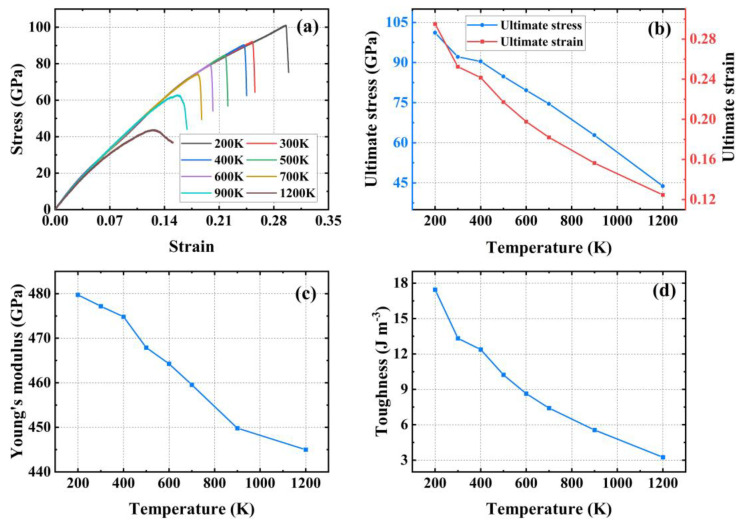
Mechanical properties of PHOTH-graphene in the armchair direction at different temperatures. (**a**) Stress–strain curve; (**b**) ultimate stress and strain; (**c**) Young’s modulus; (**d**) toughness.

Figure 8a shows the stress–strain curves of PHOTH-graphene in the armchair direction at different temperatures. The variation in ultimate stress and strain with temperature changes is illustrated in Figure 8b. As the temperature increases from 200 K to 1200 K, the ultimate stress and strain decrease by 56.69% and 57.76%, respectively. Figure 8c presents Young’s modulus at different temperatures, with values of 479.75 GPa at 200 K and 444.99 GPa at 1200 K, reflecting a decrease of 7.25%. Figure 8d shows the toughness of PHOTH-graphene at different temperatures, where an increase from 200 K to 1200 K results in a decrease in toughness from 17.45 J m^−3^ to 3.24 J m^−3^, representing an 81.43% reduction.

**Figure 9:**
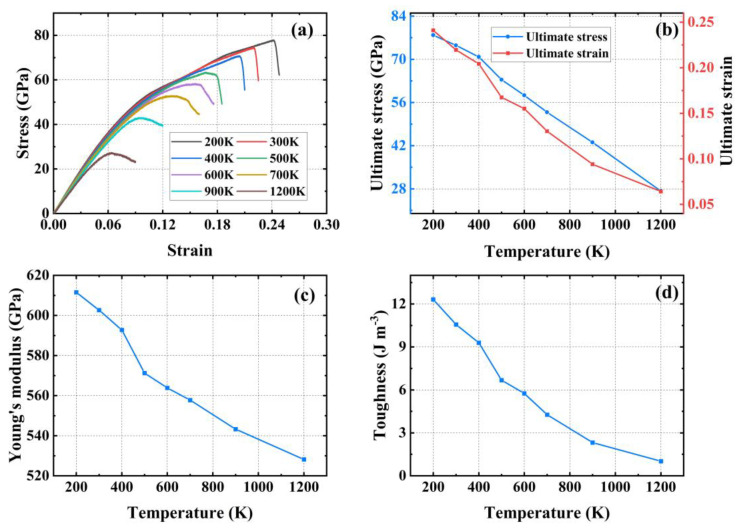
Mechanical properties of PHOTH-graphene in the zigzag direction at different temperatures. (**a**) Stress–strain curve; (**b**) ultimate stress and strain; (**c**) Young’s modulus; (**d**) toughness.

Figure 9a illustrates the stress–strain curves of PHOTH-graphene in the zigzag direction at different temperatures, showing that elevated temperature weakens the material’s strength. Figure 9b shows the ultimate stress and strain at different temperatures: 77.85 GPa and 0.24 at 200 K, and 27.33 GPa and 0.06 at 1200 K. Figure 9c presents the Young’s modulus at different temperatures: 611.52 GPa at 200 K and 528.15 GPa at 1200 K. Figure 9d shows the toughness of the material at different temperatures: 12.32 J m^−3^ at 200 K and 1.01 J m^−3^ at 1200 K. The increase in temperature from 200 K to 1200 K results in decreases in Young’s modulus, toughness, ultimate stress, and strain by 13.63%, 91.80%, 64.89%, and 75.00%, respectively.

**Figure 10:**
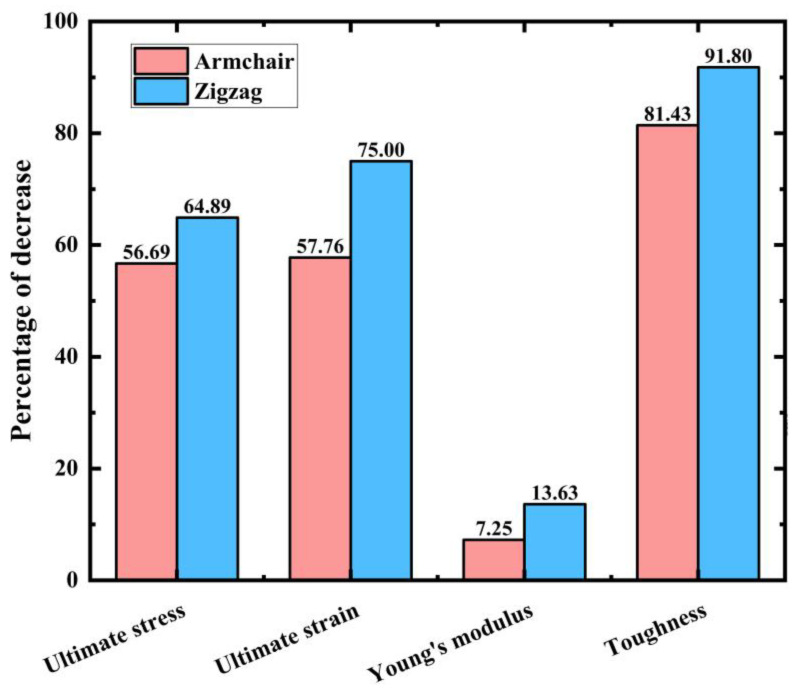
Percentage decrease in ultimate stress, ultimate strain, Young’s modulus, and toughness after temperature increases from 200 K to 1200 K.

Figure 10 shows the percentage decrease in the mechanical properties of PHOTH-graphene in both directions after increasing the temperature by 1000 K (from 200 K to 1200 K). The comparison indicates that the mechanical properties of PHOTH-graphene in the zigzag direction are more sensitive to temperature. Young’s modulus is significantly less sensitive to temperature than toughness, and ultimate stress and strain.

The C-C bond is stretched until it breaks as a stress-activated process [60]. The relationship between fracture strength, strain rate, and temperature can be described by the Arrhenius equation [15]:

(7) $$\varepsilon =A\sigma^{\frac{1}{m}}exp(-\frac{Q}{RT})$$

where ε—strain rate, A—indexing factor, m—sensitive factor, R—general gas constant, T—temperature, Q—activation energy, and σ—breaking strength.

Equation (7) shows that keeping the strain rate constant during uniaxial stretching decreases fracture strength at elevated temperatures. The higher temperature increases the vibration and kinetic energy of the atoms, which leads to more drastic changes in the bond lengths of the atomic bonds, weakened interatomic interaction forces, and a softer material that becomes more susceptible to deformation, which ultimately causes a decrease in the strength of the material [70,71].

### 3.4. Effect of Defects

#### 3.4.1. Random Vacancy Defects

Two-dimensional carbon materials inevitably develop defects during manufacturing and engineering applications, with vacancy defects being the most common. To explore the influence of random vacancy defects, we conducted in-plane mechanical property tests on materials without defects (0%) and with five concentrations of random vacancy defects: 1%, 2%, 3%, 4%, and 5%. Each concentration was tested three times. The PHOTH-graphene system remained stable after introducing the defects.

**Figure 11:**
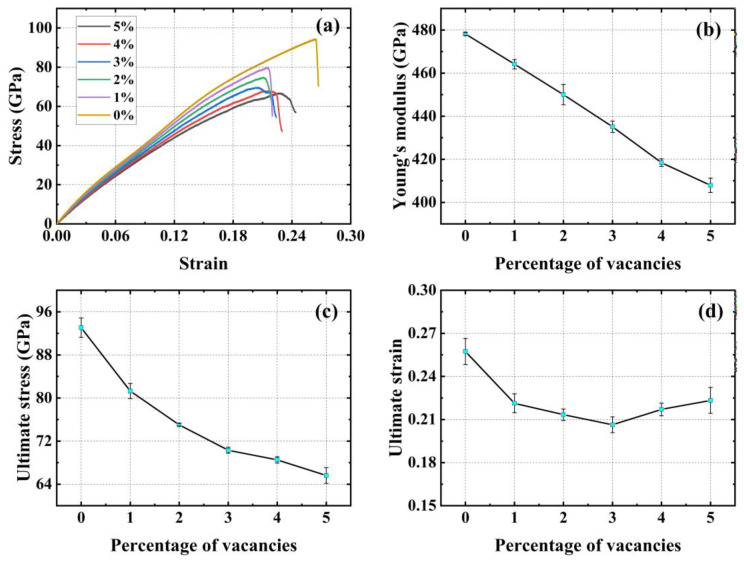
Mechanical properties of PHOTH-graphene in the X (armchair) direction for different random vacancy defect concentrations. (**a**) Stress–strain curve; (**b**) Young’s modulus; (**c**) ultimate stress; (**d**) ultimate strain.

Figure 11a shows the stress–strain curves of the material with different concentrations of random vacancy defects in the X (armchair) direction. The addition of these defects reduces the strength of the material. Figure 11b demonstrates a monotonic decrease in Young’s modulus with increasing concentration of vacancy defects. Figure 11c demonstrates the monotonically decreasing trend of the ultimate stress with increasing concentration of vacancy defects. It is noteworthy that the ultimate strain of the material initially decreases and then increases as the concentration of vacancy defects rises. As shown in Figure 11d, the ultimate strain of the material monotonically decreases and the ultimate strain decreases by 19.86% when the vacancy defect concentration increases from 0% to 3%, while the ultimate strain of the material monotonically increases and the ultimate strain increases by 8.25% when the vacancy defect concentration increases from 3% to 5%. In summary, the mechanical properties of PHOTH-graphene in the armchair direction show a decrease in the ultimate stress, ultimate strain, and Young’s modulus of the material with the addition of different proportions of random vacancy defects.

**Figure 12:**
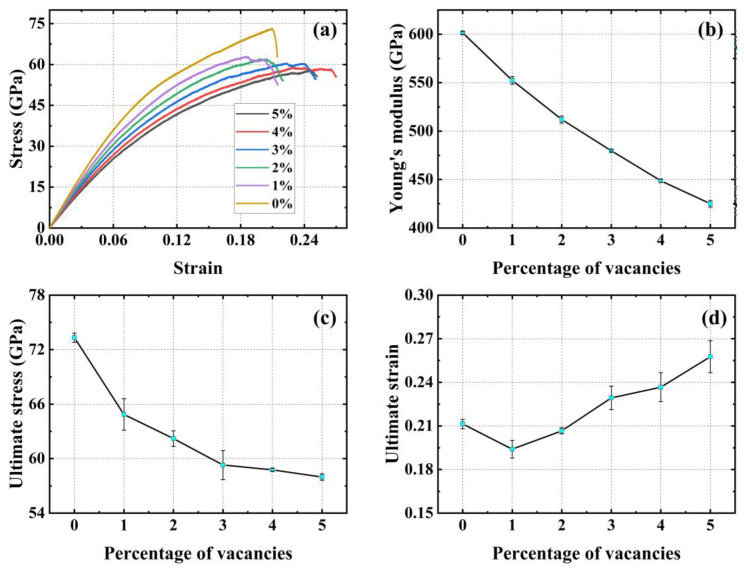
Mechanical properties of PHOTH-graphene in the Y (zigzag) direction for different random vacancy defect concentrations. (**a**) Stress–strain curve; (**b**) Young’s modulus; (**c**) ultimate stress; (**d**) ultimate strain.

Figure 12a illustrates the stress–strain curves for uniaxial stretching with the addition of different vacancy defect concentrations in the Y (zigzag) direction. Figure 12b,c demonstrate the monotonic decrease in the Young’s modulus and ultimate stress with an increase in the vacancy defect concentration, respectively. Figure 12d demonstrates the trend of the ultimate strain of the material with an increasing concentration of vacancy defects; compared to the material without added vacancy defects, the ultimate strain of the material with 1% and 2% concentration of vacancy defects decreases by 8.06% and 1.89%. The ultimate strain of the material with 3%, 4%, and 5% concentration of vacancy defects increases by 8.53%, 12.32%, and 22.27%.

**Figure 13:**
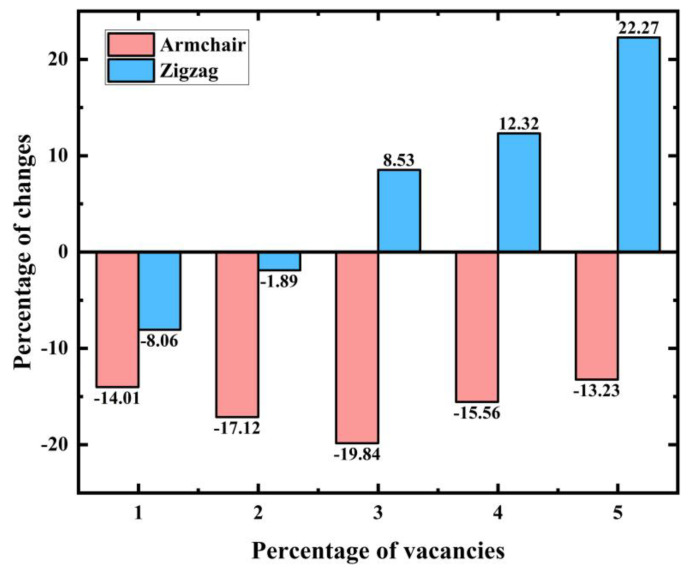
Percentage change in ultimate strain with different percentages of random vacancy defects. The percentage of random vacancy defects increased from 1% to 5%.

It is noteworthy that the ultimate strain of the material did not decrease monotonically with increasing percentage of random vacancy defects, and Figure 13 further compares the changes in the ultimate strain of PHOTH-graphene in the armchair and zigzag directions with increasing percentage of random vacancy defects. In the armchair direction, the addition of random vacancy defects causes the ultimate strain of the material to decrease, and the percentage of random vacancy defects after 3% of the ultimate strain no longer decreases but rather increases again. In the zigzag direction, the addition of 1% and 2% of random vacancy defects causes the ultimate strain of the material to decrease, and further increasing the percentage of random vacancy defects to 3%, 4%, and 5% increases the ultimate strain, which improves the strength of PHOTH-graphene to some extent. At random vacancy defect concentrations below 3%, the ultimate strain in both directions was reduced for PHOTH-graphene with added random vacancy defects compared to the material without added defects. However, at random vacancy defect concentrations higher than 3%, the ultimate strain in the zigzag direction was instead greater for the material with added defects than for the material without added defects.

#### 3.4.2. Type I Cracks and Crack Tip Hydrogen Absorption

**Figure 14:**
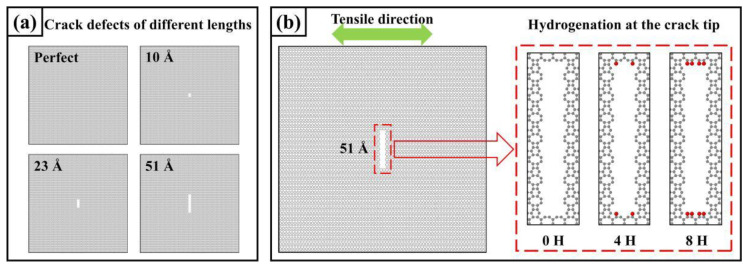
Models for hydrogen absorption at the crack tip and cracks. (**a**) Models for crack defects with different lengths; (**b**) Models for hydrogen absorption at the crack tip.

Cracks affect the mechanical properties of materials, with type I cracks being the most common form of cracks causing fracture failure of materials. As shown in Figure 14a, in this paper, type I cracks with lengths of 10 Å, 23 Å, and 51 Å are further considered on the basis of a model without defects. The width of the crack is 8 Å. The model remains stable during model relaxation, and the edge structure of the crack does not change.

As shown in Figure 14b, to further explore the influence of hydrogen adsorption at the crack tip on the material’s strength, we considered three different crack tips with a length of 51 Å—0H, 4H, and 8H, indicating 0, 4, and 8 hydrogen atoms adsorbed at the crack tip, respectively. The direction of uniaxial stretching is labeled in the figure. It should be noted that the adsorption of hydrogen atoms at the crack tip is all in-plane adsorption. This study preliminarily investigates the effect of hydrogen atom adsorption at the crack tip without exploring all possible adsorption sites or numbers of hydrogen atoms.

**Figure 15:**
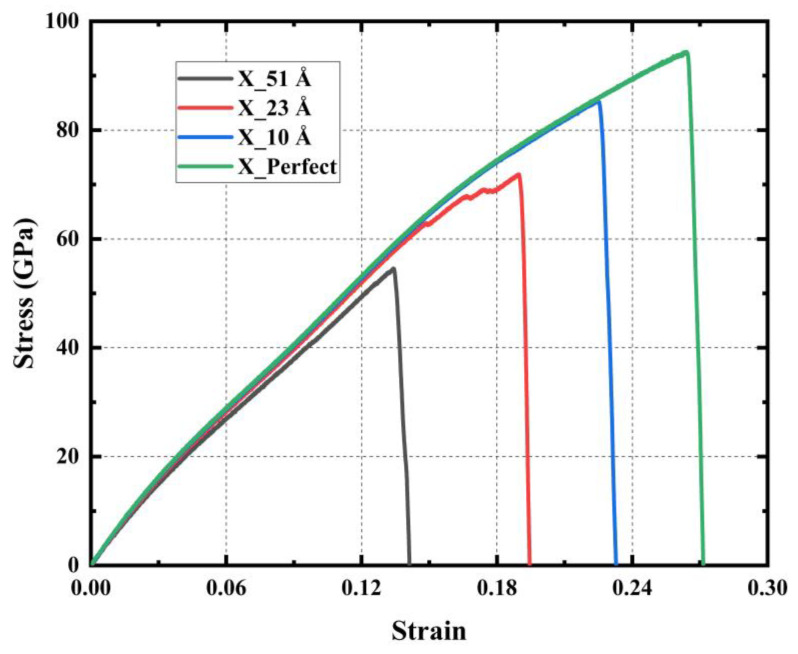
Stress–strain curves of models with different length cracks under uniaxial tensile action.

Figure 15 shows that cracks of different lengths weaken the strength of PHOTH-graphene to varying degrees. Compared with the model without cracks, the cracks with lengths of 10 Å, 23 Å, and 51 Å reduced the Young’s modulus by 0.15%, 1.24%, and 3.46%; the ultimate stress by 9.48%, 23.84%, and 42.16%; and the ultimate strain by 14.77%, 28%, and 28%, respectively. The ultimate stresses were reduced by 9.48%, 23.84%, and 42.16%; the ultimate strains were reduced by 14.77%, 28.42%, and 49.24%; and the toughness was reduced by 25.12%, 46.43% and 73.07%, respectively. Young’s modulus is obviously less sensitive to crack length changes than ultimate stress, strain, and toughness. Figure 17a shows the von Mises stress distribution during uniaxial stretching of a material with a crack length of 51 Å. An apparent stress concentration occurs at the crack tip, causing the material to fail preferentially at this location. Further extension of the crack ultimately leads to the material’s fracture failure.

**Figure 16:**
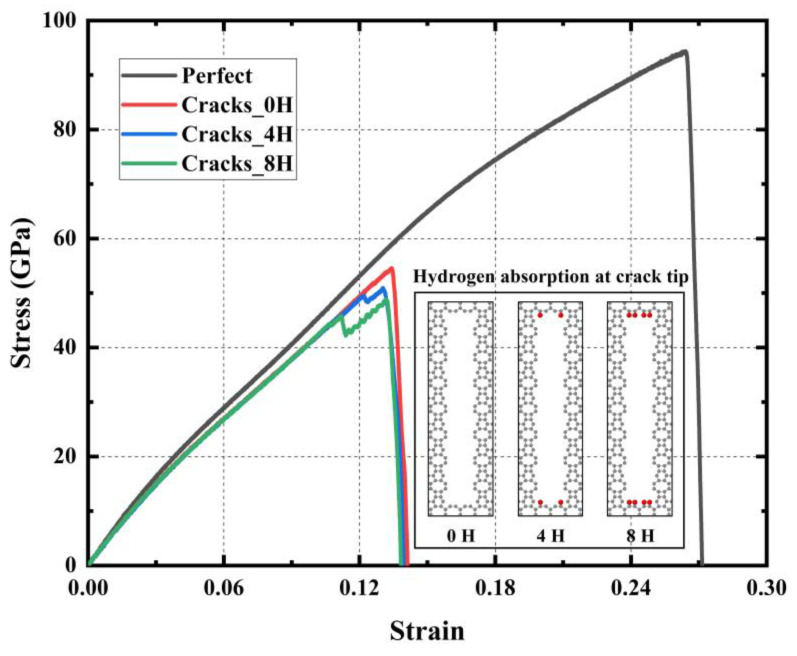
Stress–strain curves of models with different crack tips under uniaxial tensile action.

Figure 16 shows the stress–strain curves under uniaxial tensile action for models with different crack tips, each with a crack length of 51 Å. The adsorption of hydrogen atoms at the crack tip somewhat reduces the material’s mechanical properties. Compared to crack tips with no hydrogen atoms, crack tips with four and eight hydrogen atoms result in reductions in Young’s modulus by 0.22% and 0.30%, ultimate stress by 6.67% and 10.46%, ultimate strain by 2.99% and 1.49%, and toughness by 6.19% and 5.93%, respectively. The value of Young’s modulus is insensitive to hydrogen adsorption at the crack tip and hardly changes after adsorption.

Table 2 lists the values of the mechanical properties under uniaxial tension for a perfect material without crack defects, a material with three different crack sizes, and a model with three different crack tips.

**Figure 17:**
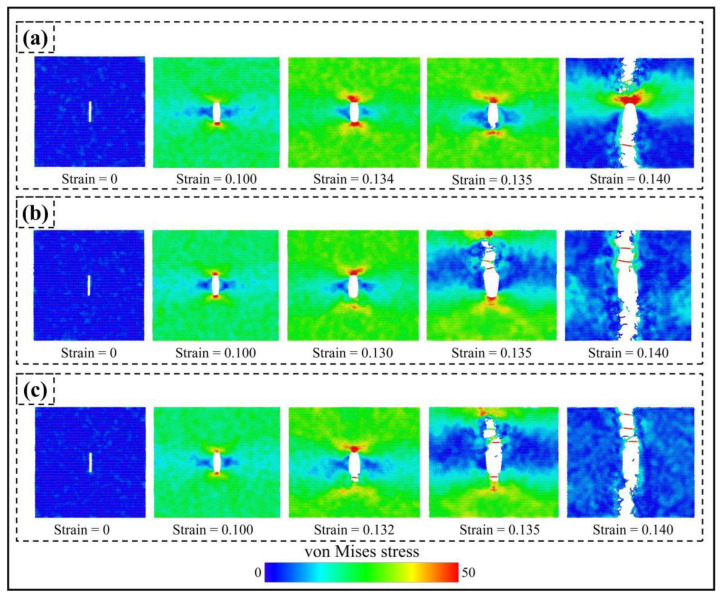
Von Mises stresses during fracture failure for models with three different crack tips: (**a**) no hydrogen atoms adsorbed at the crack tip; (**b**) four hydrogen atoms adsorbed at the crack tip; (**c**) eight hydrogen atoms adsorbed at the crack tip. The crack lengths are all 51 Å.

Figure 17 illustrates the von Mises stress distribution during fracture for cracks with three different crack tips, all exhibiting significant stress concentrations at the crack tip. At a strain of 0.135, the model crack with hydrogen atoms adsorbed at the crack tip expands rapidly, while the crack with no hydrogen atoms begins to form. Hydrogen atoms cause the crack to expand at a much smaller strain because the strength of the C-C bonds at the hydrogen adsorption sites is weakened [33,36,66,67,72]. Thus, hydrogen atom adsorption at the crack tip further weakens the material’s mechanical properties.

## 4. Conclusions

This study tested the mechanical properties of a new two-dimensional carbon material, PHOTH-graphene, using an optimized REBO potential function based on a classical molecular dynamics approach. The following conclusions were obtained:

(1) PHOTH-graphene exhibits excellent mechanical properties in both the armchair and zigzag directions, but there are also significant differences. The material has stronger ultimate stress, ultimate strain, and toughness in the armchair direction and greater Young’s modulus in the zigzag direction.
(2) The failure fracture patterns of PHOTH-graphene differ between the armchair and zigzag directions. Microcracks are first observed when the material is stretched uniaxially in the armchair direction, and regions of the amorphous structure appear in the direction of crack tip expansion. Finally, brittle fracture occurs as the crack expands. The region containing the amorphous structure is observed first when the material is stretched uniaxially in the zigzag direction; then, microcracks appear in the neighborhood of the amorphous region; and the material fails due to fracture as the crack expands.
(3) Increasing the temperature weakens the strength of PHOTH-graphene. An increase in temperature by 1000 K (from 200 K to 1200 K) results in varying temperature sensitivities of the material’s mechanical properties in both directions. Young’s modulus is less sensitive to temperature increases than toughness, ultimate stress, and strain.
(4) The introduction of random vacancy defects significantly weakened the strength of PHOTH-graphene. The values of ultimate stress, Young’s modulus, and toughness all decreased monotonically with increasing concentrations of random vacancy defects. Notably, increasing the concentration of random vacancy defects can enhance the ultimate strain of the material. Particularly, after increasing the concentration of vacancy defects by 3% in the zigzag direction, the ultimate strain of the material is higher than that of the material with no vacancy defects.
(5) The stress concentration area during uniaxial stretching appears at the tip of a type I crack, significantly weakening the mechanical properties of PHOTH-graphene. The adsorption of hydrogen atoms at the crack tip induces the crack to expand under more minor strains, further weakening the material’s strength.

## Figures and Tables

**Table 1:** Mechanical properties of PHOTH-graphene in the zigzag and armchair directions.

Tensile Direction	Ultimate Stress (GPa)	Ultimate Strain	Young’s Modulus (GPa)	Toughness (J m^−3^)
Along zigzag	73.14	0.211	602.16	10.56
Along armchair	94.38	0.264	477.38	14.41

**Table 2:** Mechanical properties of PHOTH-graphene with cracks.

Type of Crack	Ultimate Stress (GPa)	Ultimate Strain	Young’s Modulus (GPa)	Toughness (J m^−3^)
Perfect	94.38	0.264	477.38	14.41
10 Å	85.43	0.225	476.65	10.79
23 Å	71.88	0.189	470.39	7.72
51 Å	54.59	0.134	461.88	3.88
51 Å_4H	50.95	0.130	460.87	3.64
51 Å_8H	48.88	0.132	460.50	3.65

## Data Availability

The original contributions presented in the study are included in the article, further inquiries can be directed to the corresponding authors.
